# A longitudinal cohort of stress cardiomyopathy assessed with speckle-tracking echocardiography after moderate to severe traumatic brain injury

**DOI:** 10.1186/s13054-020-02935-1

**Published:** 2020-05-12

**Authors:** Raphaël Cinotti, Thierry Le Tourneau, Kalyane Bach-Ngohou, Maxime Le Courtois du Manoir, Bertrand Rozec, Karim Asehnoune

**Affiliations:** 1 Anesthesia and Critical Care Department, Hôpital Guillaume et René Laennec, University Hospital of Nantes, Boulevard Jacques Monod, 44800 Saint-Herblain, France; 2 Institut du Thorax, INSERM UMR1087, University Hospital of Nantes, Nantes, France; 3 Department of Biochemistry, INSERM U1235, Hôtel Dieu, University Hospital of Nantes, 1 place Alexis Ricordeau, 44093 Nantes Cedex 9, France; 4 Anesthesia and Critical Care Department, Hôtel Dieu, University Hospital of Nantes, 1 place Alexis Ricordeau, 44093 Nantes Cedex 9, France; 5 Laboratoire UPRES EA 3826 “Thérapeutiques cliniques et expérimentales des infections”, University Hospital of Nantes, 1 rue Gaston Veil, 44035 Nantes Cedex 1, France

**Keywords:** Stress cardiomyopathy, Traumatic brain injury, Speckle-tracking echocardiography

## Research letter

Stress cardiomyopathy is common after subarachnoid haemorrhage (SAH): 36% of patients display stress cardiomyopathy patterns assessed with speckle-tracking echography [1], which is a gold standard in the evaluation of left ventricular longitudinal systolic function. After traumatic brain injury (TBI), stress cardiomyopathy has been little described [2]. We performed a monocentric longitudinal study in moderate to severe TBI patients (Glasgow coma score ≤ 12). Consecutive patients were included. This study was approved by the local ethics committee (Groupe Nantais d’Ethique dans le Domaine de la Santé – IRB No. 6.02.2014). We a priori decided to include 100 patients in order to potentially detect 30 patients with sub-clinical stress cardiomyopathy [1]. The primary goal was to assess the incidence of stress cardiomyopathy with speckle-tracking echocardiography and the evolution of the global longitudinal strain (GLS) at day 1, day 3, and day 7. The secondary outcomes were the evolution of 2-dimensional echocardiographic parameters (LVEF, mitral E/A and E/E’ ratio, mitral S wave, TAPSE). Since stress cardiomyopathy is due to a major catecholamine increase in plasma [3], we explored the adrenergic response by comparing baseline blood levels of metanephrine and normetanephrine in patients with TBI and SAH admitted in our institution, matched on age and baseline GCS (biocollection IBIS – NCT 02426255). We included 100 patients from March 2014 to August 2017. The mean age was 42.6 (± 19.6) years and the baseline Glasgow coma score was 7 [4–10]. We included 75 (75%) male and 25 (25%) female patients. Twenty (20%) patients died in the ICU. At day 1, GLS (− 20.3 [± 3.6]%) and LVEF (66 [± 11]%) were preserved. The mean GLS was preserved at day 3 (− 22.2 [± 3.6] %) and at day 7 (− 20.7 [± 3.3] %). Nine (9%) patients displayed impaired GLS (− 13.3[− 14.5; − 11.6]%) at baseline. In these patients, there was a significant improvement at day 3 (− 22.2 [− 25.1; − 18.7]%) and day 7 (− 21.1 [− 23.2; − 18.1]%) (*p* < 0.0001), compatible with stress cardiomyopathy. These 9 patients had the same age (32 [23–48] vs 46 [23–60], *p* = 0.4), had a non-significant baseline ultra-sensitive troponin increase (16 [8–229] vs 9 [5–29] ng/mL^−1^, *p* = 0.1), and had similar Glasgow (10 [3–12] vs 7 [4–9], *p* = 0.3) and Marshall scores (*p* = 0.8) compared to the rest of the cohort. Three patients suffered from isolated TBI, and two from TBI associated with mild abdominal trauma or vertebral fracture, all due to road traffic accidents. The remaining four patients suffered from isolated TBI after a fall. These mechanisms did not seem to differ from the rest of the cohort.

**Fig. 1:**
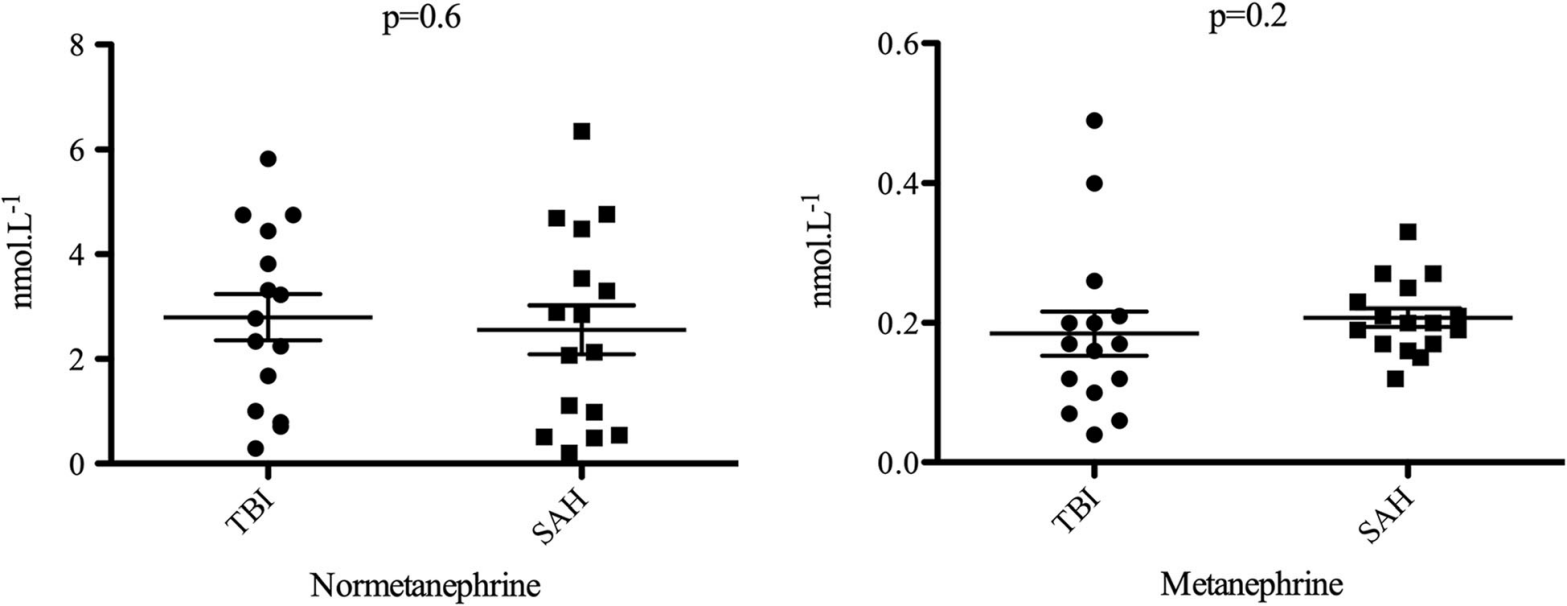
Baseline blood levels of metanephrine and normetanephrine in 30 patients with TBI or SAH admitted in a single institution

In the overall cohort, right ventricular TAPSE at day 1 was preserved (21.6 (± 7.6) mm) and significantly improved at day 3 (24.8 (± 5.3) mm, *p* = 0.003). There was no significant modification of LVEF, the E/A and E/E’ ratios, or lateral S wave. In order to assess the adrenergic response, we measured baseline metanephrine and normetanephrine blood levels in 15 SAH and 15 TBI patients. There was no significant difference in normetanephrine (2.5 [0.7–4.2] nmol/L^−1^ vs 2.9 [1–4.4] nmol/L^−1^, *p* = 0.6) and metanephrine (0.2 [0.17–0.23] nmol/L^−1^ vs 0.17 [0.1–0.21] nmol/L^−1^, *p* = 0.2) plasma levels between SAH and TBI patients (Fig. 1), which challenges the adrenergic response as the only trigger for stress cardiomyopathy. However, the plasma levels were not measured just after the onset of brain injury, and considering that the catecholamine levels may rapidly change over time along with the modest sample size, we cannot ascertain that blood levels are comparable between TBI and SAH patients.

Stress cardiomyopathy occurs after traumatic brain injury, recovers promptly, but is less common (≈ 10%) than after SAH (≈ 35%). The raised baseline metanephrine and normetanephrine was comparable in our sample of TBI and SAH patients. Sympathetic hyperactivation is perhaps not the only mechanism involved in stress cardiomyopathy.

## Data Availability

The datasets used and/or analysed during the current study are available from the corresponding author on reasonable request.

## Abbreviations

TBI: Traumatic brain injury
GLS: Global longitudinal strain
TTE: Trans-thoracic echocardiography
SAH: Aneurysmal subarachnoid haemorrhage
LVEF: Left ventricular ejection fraction
LV: Left ventricle
ICU: Intensive care unit
GCS: Glasgow Coma Score
TAPSE: Tricuspid annular plane systolic excursion
